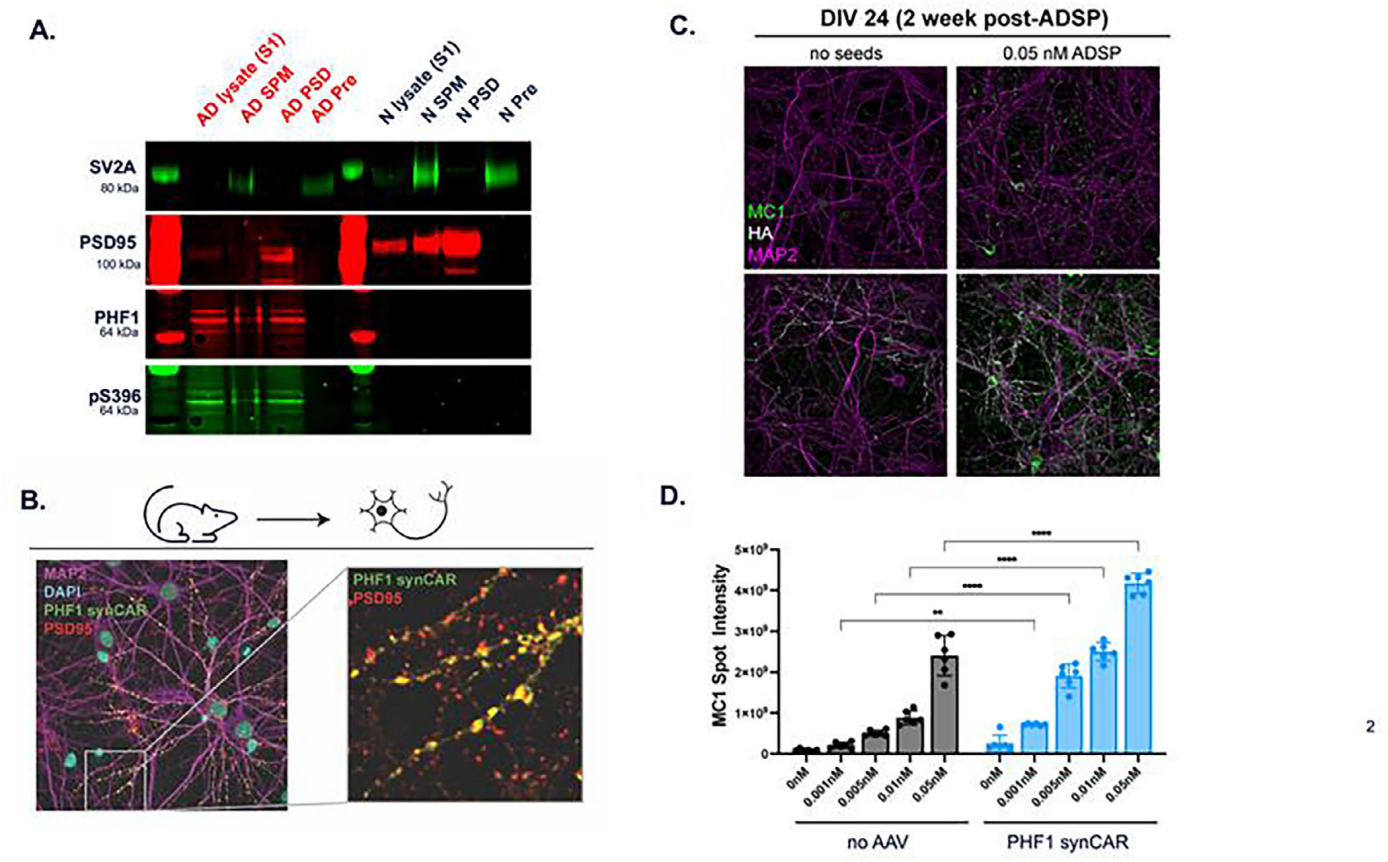# Selective targeting of trans‐synaptic tau using chimeric antibodies receptors tethered to the synaptic membrane

**DOI:** 10.1002/alz70855_103576

**Published:** 2025-12-24

**Authors:** Jessica Wu, Rebecca Sebastian, Robert Kupp, Dhaval Nanavati, Iwan Parfentev, Nandini Romanul, Kiran Yanamandra, Christina Preiss, Justine Manos, Haiyan Wu, Sharmin Afroz, Jonathan A Sreter, Wendy Ritacco, Lionel M Galibert, Alessandra M Welker, Xavier Langlois

**Affiliations:** ^1^ AbbVie, Cambridge, MA, USA; ^2^ AbbVie, San Francisco, CA, USA; ^3^ AbbVie, Worcester, MA, USA; ^4^ AbbVie, Ludwigshafen, Ludwigshafen, Germany; ^5^ AbbVie, Cambridge Research Center, Cambridge, MA, USA; ^6^ AbbVie Inc. Foundational Neuroscience Center, Cambridge, MA, USA

## Abstract

**Background:**

Trans‐synaptic transmission of pathological tau contributes to the spread of tau aggregates and neurofibrillary formation in Alzheimer's disease (AD). Reducing the prevalence of pathogenic tau species within the synaptic cleft may therefore prevent tau propagation and attenuate AD progression. Passive immunotherapy using monoclonal IgG antibodies has been used to target extracellular tau, however these therapies have not achieved clinical success suggesting a failure to target propagating tau species. Additional challenges such as antibody selectivity towards spreading species, availability within the CNS and physical limitations in accessing the narrow synaptic cleft may further prevent immunotherapies from accessing and identifying trans‐synaptic propagating tau.

**Method:**

To address these limitations, we purified synaptosomes from AD and pathologically negative control patients and used phospho‐proteomics to identify tau epitopes present in the AD post‐synaptic terminals. Comparison of the AD and normal post‐synaptic fractions identified 17 phospho‐tau epitopes enriched in the AD fraction, including the PHF1 tau epitope pS396/pS404.

**Result:**

Using this information, we developed a novel approach to increase effective anti‐tau antibody concentration at the synapse by using AAV9 to deliver a tethered synaptic‐chimeric antibody receptor (synCAR) to the post‐synaptic membrane. To achieve this aim, we fused the PHF1 small antibody fragment (scFv) with the post‐synaptic membrane component neurolignin‐1 (NLGN1). Using both murine and hiPSC neuronal models, we show that our PHF1 synCAR effectively expresses at the synaptic membrane and localizes with the post‐synaptic marker PSD95 without impacting neuronal viability. Finally, we show that PHF1 synCAR effectively targets pathogenic tau species by observing that tau aggregation in seeded mouse primary neurons increased by ∼73%. This indicates that PHF1 synCAR captures and concentrates tau seeds resulting in accelerated seeding pathology.

**Conclusion:**

Overall, these data suggest a novel mechanism for targeting proteins either in the synaptic cleft or within the post‐synaptic membrane and provides an effective approach for isolating and identifying propagating tau species.

AbbVie Disclosure Statement:

All authors are employees of AbbVie. The design, study conduct, and financial support for this research were provided by AbbVie. AbbVie participated in the interpretation of data, review, and approval of the publication. No honoraria or payments were made for authorship.